# The development and psychometric properties of a measure to assess the written submission of an admissions application

**DOI:** 10.1177/03080226221080871

**Published:** 2022-04-22

**Authors:** Jill Stier, Jill Cameron, Behdin Nowrouzi-Kia, Chantel Brammer, Sara Asher, Deborah Lipszyc

**Affiliations:** 1 Department of Occupational Science and Occupational Therapy, 12366 University of Toronto, Toronto, ON, Canada; 2 Department of Occupational Science and Occupational Therapy, 12366 University of Toronto, Toronto. Canada 8613 North York General Hospital, Toronto, Canada; 3 508783 St. Joseph’s Health Centre, Unity Health Toronto, Toronto, Canada; 4 Department of Family and Community Medicine, 12366 University of Toronto, Toronto, Canada

**Keywords:** admission criteria, instrument development, occupational therapy, measurement

## Abstract

**Introduction:**

Health care programs evaluate prospective applicants using cognitive and non-cognitive criteria. The aim of this research was to develop and evaluate the psychometric properties of a measure to evaluate the non-cognitive criteria of admissions applications.

**Method:**

A Masters of Occupational Therapy Written Submission Measure (MOTWSM) was developed and evaluated over 3 phases, using applicants’ written statements, resumes, and reference letters. Participants included 50 students who completed an occupational therapy program for determination of internal consistency and test-retest reliability. Additionally, 195 written submissions selected from the applicants who were admitted, waitlisted, and not admitted to the program were evaluated to determine inter-rater reliability using a two-way ANOVA. Analysis of 195 submissions using a one-way ANOVA determined the measure’s discriminative validity.

**Findings:**

Results indicated test-retest reliability of 0.95 and internal consistency reliability of 0.76. Inter-rater reliability reported a Cronbach’s alpha coefficient of 0.86 using a horizontal scoring method. Good discriminative validity was established.

**Conclusion:**

The MOTWSM is a reliable and valid measure that can be used to evaluate the non-cognitive criteria of admissions applications in health profession programs. Use of this measure can facilitate selection of the highest caliber of students.

## Introduction

Graduate programs in Occupational Therapy aim to produce graduates who develop the knowledge and professional competencies essential for practice (Lyons et al., 2006). Admission committees have the important task of identifying the applicants most suited for admittance into health professional programs, as these students will impact the future of healthcare (Lyons et al., 2006; Unni et al., 2011; Urlings-Strop et al., 2013). In order to uphold the standards of these professions, their respective associations, the public, and admission committees must ensure that their procedures are efficient, reliable, and valid (Salvatori, 2001; Siu and Reiter, 2009) through evidence based guidelines.

Admission committees most commonly utilize cognitive criteria such as pre-admission grade point average (pre-GPA), and the Graduate Record Exam (GRE) to predict academic excellence (Coates, 2008; Salvatori, 2001). Pre-GPA is the best predictor of academic success and is one of the most heavily weighted admission components (Lysaght et al., 2009; Salvatori, 2001). There is evidence of predictive validity of pre-GPA on academic performance in many graduate and undergraduate professional disciplines, including medicine, dentistry, nursing, physical therapy, and occupational therapy (Kirchner and Holm, 1997; Kirchner et al., 2001; Lysaght et al., 2009; Salvatori, 2001). Specific to occupational therapy, Lysaght et al. (2009) examined the predictive value of selected pre-admission criteria relative to student outcomes at a Canadian University occupational therapy graduate program. They concluded that a high pre-GPA was significantly correlated with a high overall student academic average. While cognitive criteria are commonly used in the admissions process, they are often utilized in conjunction with one or more non-cognitive criteria.

Non-cognitive criteria (interviews, multiple mini interviews, written statements, resumes, and reference letters) (Auriemma, 2007; Brown et al., 1991) are routinely used to evaluate admission packages in many health profession programs. There is strong support for the utilization of these components because of their ability to predict future performance in professional practice (Lyons et al., 2006; Salvatori, 2001). Interviews are commonly used to evaluate the non-cognitive elements of applicants’ admission packages such as the personal traits and qualities of applicants; however, there is variability in the reliability and validity of these measures (Dore et al., 2006; Gabard et al., 1997; Gafni et al., 2003; Kreiter et al., 2004; Lyons et al., 2006; McNelis et al., 2010; Salvatori, 2001). Additionally, interviews lack standardization and the ability to predict successful completion of professional programs (Gabard et al., 2006; Kreiter et al., 2004).

A commonly used non-cognitive criteria is written statements. Written statements require applicants to respond to specific open-ended questions to evaluate their experience, goals and fit for the profession. Research supports written statements in the admissions process as they contain pertinent information concerning the applicant’s qualities, values, and motivation required for a healthcare profession (Kirchner and Holm, 1997). There can be many advantages to using written statements because a variety of practice competencies can be evaluated including conscientiousness, emotional intelligence (Gutman, S, and Falk-Kessler, 2018; MacCann et al., 2020), and empathy (Thomas et al., 2017). Despite these advantages, the psychometric properties related to the evaluation of written statements have been inconsistent and would benefit from effective guidelines to ensure admission decisions are evidence based. The measures used to evaluate these statements tend to be unstructured and vary from program to program. Brown, Carpio, and Roberts (1991) suggested that the reliability and validity of autobiographical essays could be improved by utilizing a standardized Likert scale. Despite limited and conflicting evidence on the psychometric properties of written statement measures, their use helps programs evaluate additional characteristics, such as fit and the knowledge of the specific health profession which are not adequately identified by other non-cognitive criteria.

Reference letters are another commonly used non-cognitive element that may identify personal qualities and traits essential for professional practice, which can be similar across a variety of health professions. Lysaght, Donnelly, and Villeneuve (2009) found a positive correlation between referee ratings and occupational therapy students’ interpersonal communication skills. However, there is variability in letters written by different referees, specifically, clinical supervisors or teaching faculty (Stedman et al., 2009). Further evidence indicates that reference letters did not consistently predict medical school performance (Patterson et al., 2016). Standardized evaluation guidelines are recommended to enhance the evaluation of reference letters (Dirschl and Adams, 2000; Nayer, 1992). Despite varying evidence on the evaluation of reference letters, health profession programs tend to continue to require them as part of the admissions package.

It is also important to consider the different scoring methods used to rate written application packages. A common evaluation method used by admissions committees is the vertical scoring method. The vertical scoring method evaluates all components of one application before evaluation of the next application. However, use of the vertical scoring method has been shown to produce the halo effect, which decreases the reliability and validity of the scores (Dore et al., 2006). The halo effect occurs when a rater’s score on one component of an application influences the rater’s perception and score of subsequent components (Dore et al., 2006). Research has shown that the halo effect can be minimized through use of the horizontal scoring method (Dore et al., 2006; Peeters et al., 2015). The horizontal scoring method entails evaluating one component across all applicants before evaluating the next component (Dore et al., 2006). Based on the evidence, implementation of the horizontal scoring method into admissions procedures may be a viable method to improve the reliability and validity of the rated scores.

To our knowledge, there are no psychometrically sound instruments available to evaluate written assessments of non-cognitive criteria. The purpose of this research is to describe the development of the Masters of Occupational Therapy Written Submission Measure (MOTWSM) used to evaluate non-cognitive elements of admissions applications and to determine the measure’s psychometric properties using a horizontal method for evaluation. It is crucial that a psychometrically sound scale is developed and utilized to assess non-cognitive criteria so that the most suitable applicants gain entrance into undergraduate or graduate occupational therapy programs.

## Methods

### Design

Development and validation of the MOTWSM occurred in three phases and utilized steps of instrument development consistent with Kyriazos & Stalikas approach (2018). Phase 1: MOTWSM instrument development included the definition of its purpose, identification of the scale format, item generation, questionnaire formatting, and consultation with an expert panel regarding item selection. Phase 2: assessment of usability, reliability (internal consistency, test-retest) and construct validity (convergent, divergent). Phase 3: assessment of inter-rater reliability and discriminant validity, with use of the horizontal rating method. Ethical approval was granted by the University’s research ethics board for all phases of the research.

### Phase one instrument development

#### Purpose, response scale format, item generation, item selection

A psychometrically sound measure that evaluated the non-cognitive elements of admission applications were of interest. The purpose and construct of the instrument were discussed with members of the admissions committee and experts in measurement. The admission factors already used in the program of study were discussed which included the cognitive criteria comprised of the undergraduate grade point average (pre-GPA) based on the applicants’ last full 10 credit courses. The non-cognitive criteria rated included a written submission package comprised of two written statements (responses to questions posed that articulates the candidate’s experiences, skills and knowledge of the profession), a resume and two letters of reference (one from an academic referee and one from a professional referee). Based on these criteria, items were generated to evaluate the five elements of the non-cognitive criteria, specifically the two written statements, one resume and two reference letters. These generated items were based on existing criteria used in the program of study and can be considered as valid sources of inclusion (Streiner et al., 2015). A continuous 5-point Likert scale (Streiner et al., 2015) was created at the same time as item generation to ensure congruency (Devellis, 2017). The goal was to score applicants’ profile packages using the Likert scale in a timely manner with no more than five items for evaluation of each of the two written statements, resume and reference letters.

### The masters of occupational therapy written submission measure

The MOTWSM was developed and included a 25-item scale that evaluates the five subscales of the written submission package with 5 items on a 5-point Likert scale. The time to complete the scale was recorded to ensure that it was to be completed in a timely manner (no more than 15 minutes). Each of the two written personal statement questions were evaluated to determine the extent to which the applicant displays required dimensions related to their understanding of the profession and their ability to answer the questions in a well-written, clear and comprehensive manner. The applicant’s resume was evaluated based on their participation in volunteer and leadership activities, the type of awards they have received as well as their publication history. Each of the reference letters were evaluated based on the appropriateness of the chosen referees, and their ratings and recommendations. Scores on each item range from 0 to 4, scores on each subscale range from 0 to 20, and scores for the total MOTWSM range from 0 to 100. Each item was rated from not at all (scored 0) to extremely well (scored 4). A higher score on each domain of the MOTWSM indicates a higher quality written submission.

**Figure 1.**
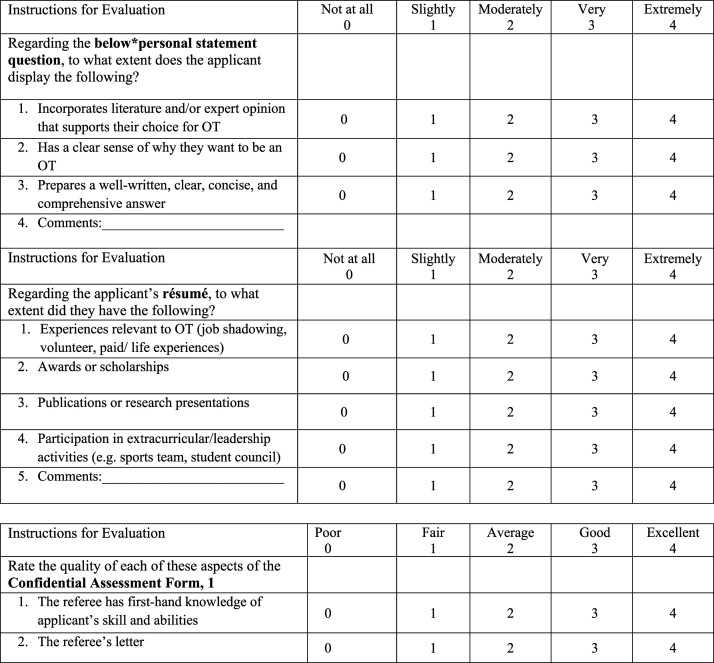
Sample items from the MScOT written submission measure (MOTWSM).

A total of 10 experts with experience on the admissions committee were given one de-identified personal profile package (applicants who were admitted and completed the program) and a copy of the newly developed MOTWSM. The panel consisted of six professors, two clinicians, one administrative staff member, and one student. Two of the professors also had expertise in measurement development. To determine content validity, the experts were instructed to score the profile package using the MOTWSM, assess the instructions, clarity and relevance of the items, response options and record the time required to score one package. These experts were familiar with rating admission packages. Previously they used an existing departmental rating system whereby trained faculty members and clinicians rated each of the two written statements on a Likert scale from 0 to 10 and the combined resume and two letters of reference using the same scale. These three scores are totaled and an overall score is created by averaging these three scores. Higher scores indicated better quality. After consultation with the ten experts, the items for evaluation were clarified based on expert comments regarding the readability of the measure. Furthermore, instructions were modified to decrease ambiguity, response options were condensed from ten to five. The scoring was modified to include separate scores for both the resume and each of the reference letters. In Figure 1, sample items are presented. All items are not presented as they may currently be used in the application process.

### Phase two instrument testing

Phase two measured the reliability and validity of the new MOTWSM. This included evaluating: (1) internal consistency; (2) test-retest reliability; and (3) construct and convergent validity.

A retrospective analysis of simple randomly sampled de-identified admissions data of 50 applicants from a Master of Science in Occupational Therapy program in Canada who were admitted to and previously completed the program was conducted using the MOTWSM. All applications were randomly selected from each group within the department database for entry class 2008, de-identified, and coded by the Graduate Administrator. Coding occurred through the deduction of one number from the applicant’s Program Application Service identification number. This enabled the raters to reallocate the written submissions to the proper group after evaluation; however, the raters were blinded to the group membership during the evaluation process. Our sample size was based on similar research conducted (Cameron et al., 2008). Six profiles had incomplete data and were not included in the analyses.

### Phase two data analysis

Internal consistency coefficient as measured by Cronbach’s alpha was calculated. Test-retest reliability was determined by re-assessing 19 profile packages after a two-week waiting period (Windsor, 2007). Construct validity was assessed by examining the correlation coefficient between the MOTWSM and pre-GPA since the latter has been established as a predictor variable for success in the Master of Science in Occupational Therapy program (Lysaght et al., 2009; Salvatori, 2001). Convergent validity was determined by examining the correlation between scores using the MOTWSM and the program’s current measure that uses a 10-point rating scale. Statistical analysis was performed with SPSS 20.0 (SPSS, 2011).

## Phase three instrument testing

**Figure 2.**
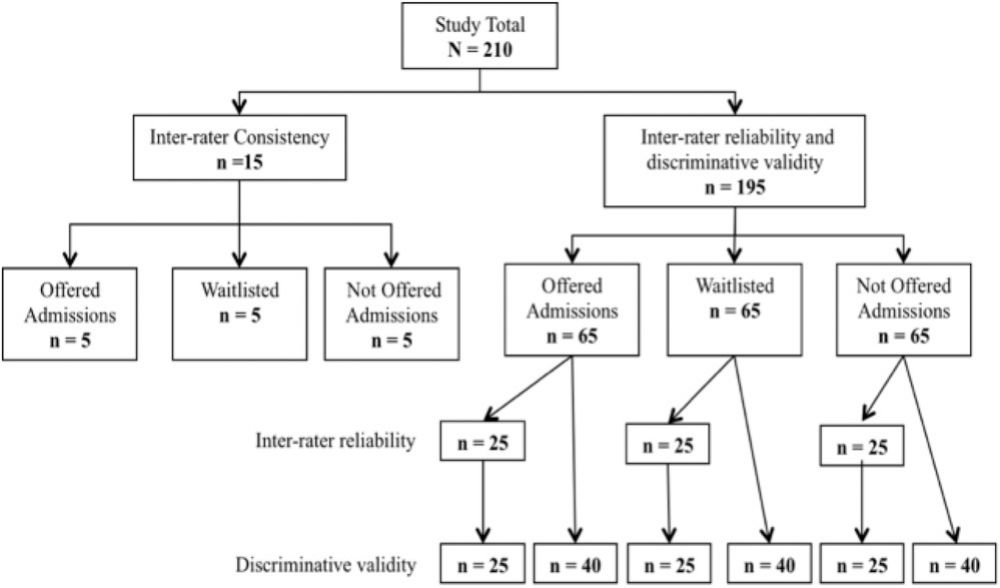
Flow chart of testing samples.

Phase three included psychometric testing using a horizontal method for evaluation. A new sample of 210 randomly selected de-identified written submission packages was evaluated. See Figure 2 for allocation of the reliability and validity testing sample of written submissions. A sample of 15 submissions, five from each of three groups who were admitted, waitlisted, or not admitted, were evaluated by two raters to identify and develop guidelines for the 5-point Likert scale for each of the 25 items on the MOTWSM. The remaining 195 written submission packages were analyzed to determine inter-rater reliability and discriminative validity with three groups of 65 applicants who were admitted, waitlisted, or not admitted. To determine inter-rater reliability, 25 written submissions were randomly selected from each of the 3 groups (admitted, waitlisted, or not admitted), for a total of 75 written submissions and each rater independently evaluated all 75 written submissions using the horizontal scoring method (Dore et al., 2006). To determine discriminative validity, 65 submissions from each of the three groups were evaluated. To reduce experimenter’s bias, the raters were blinded to the group allocation during evaluation. Throughout the evaluation process, the raters recorded the time it took to evaluate each component of the written submission with the MOTWSM using the horizontal scoring method.

### Phase three data analysis

Descriptive statistics of mean, standard deviation, and minimum and maximum values of the scores on each component and the total MOTWSM of 195 written submission packages were determined. Cronbach’s alpha was also calculated for each component and the total MOTWSM score. To determine inter-rater consistency and inter-rater reliability, the scores were compared across raters by item, component, and total MOTWSM. The ICC were determined using a two-way random effects ANOVA. An ANOVA was chosen because it enables the comparison of multiple values without the risk of a Type I error (Samuels et al., 2012). Type I errors occur when the null hypothesis that group means are equal is falsely rejected (Samuels et al., 2012). The two-way random effects ANOVA was used because the data had three factors: the first rater’s scores, the second rater’s scores, and the applicant number (Weir, 2005). Two-way random effects ANOVA also provides an ICC value, which is best practice for the determination of inter-rater reliability (McGraw and Wong, 1996; Weir, 2005). ICC values range from −1 to +1, with 0 indicating no correlation between the scores (McGraw and Wong, 1996; Samuels et al., 2012; Weir, 2005). In this study, a correlation closer to +1 indicates that the two raters have well correlated scores. To determine discriminative validity, a one-way ANOVA with linear planned contrasts was administered for the total MOTWSM scores. The contrasts consisted of admitted versus waitlisted, admitted versus not admitted, and waitlisted versus not admitted.

## Results

### Phase 1 usability

The mean length of time taken by the expert panel to score the MOTWSM was 15 min with a range of 8 min–30 min. The average time to rate 44 profiles using the MOTWSM was 12 min per profile. The expert panel deemed the measure to be user-friendly, intuitive to use, organized and easy to follow. No suggestions or improvements were recommended by the panel.

### Phase 2 reliability and validity

Guidelines for reliability coefficients as described by Law et al. (2017) report that coefficients greater than 0.80 would be rated as excellent, coefficients from 0.60 to 0.79 be rated as good or adequate and coefficients less than 0.60 be rated as low. In this study, Cronbach’s alpha coefficient of the total measure was good with a value of 0.76, suggesting that the individual items of the instrument correlated well with one another (Table 1). Removal of individual items did not result in a drop in alpha below 0.73, indicating that all items contribute relatively equally to the consistency of the scale. The subscales showed good internal consistency coefficients: Personal Statement Question Two (α = .68) and low internal consistency coefficients: Personal Statement Question One: (α = .53); Resume (α = 0.56); Letter of Reference One (α = 0.36); Letter of Reference Two (α = 0.58). The use of mean inter-item correlations as an alternative to Cronbach’s alpha was not performed as part of this study. The test-retest correlation coefficient (ICC) over a period of two weeks was 0.95, suggesting consistency between assessments. Test-retest for the subscales ranged from r = 0.88 to r = 0.96. See Table 1.

**Table 1.** ICC and internal consistency of the MOTWSM.

	Number of items	ICC 2 Week period (N=19)	Internal consistency Cronbach’s alpha (N=44)
Total Measure	25	.95	.76
PS Q1^+^ Subscale	5	.96	.53
PS Q2^++^ Subscale	5	.96	.68
Resume Subscale	5	.89	.56
LOR1^+++^ Subscale	5	.90	.36
LOR2^++++^ Subscale	5	.88	.58

Note: ^+^ PS Q1- Personal Statement Question 1

^++^PS Q2-Personal Statement Question 2

^+++^LOR1- Letter of Reference 1

^++++^LOR2- Letter of Reference 2

**Table 2.** Correlation matrix.

	1	2	3	4	5	6	7	8
1. MOTWSM	-	.69***	.77***	.77***	.45***	.45***	.55***	.23
2. PS Q1		-	.55***	.47***	.13	.01	.38**	.05
3. PS Q2			-	.55***	.04	.13	.36**	.32**
4. Resume				-	.13	.06	.28	.26*
5. LOR1					-	.40***	.35**	−.02
6. LOR2						-	.41***	.03
7. Current measure							-	.03
8. Pre-GPA								-

Note: *p < 0.1 **p < 0.05, ***p < 0.01.

Note: PS Q1- Personal Statement Question 1

PS Q2-Personal Statement Question 2

LOR1- Letter of Reference 1

LOR2- Letter of Reference 2

Table 2 lists the correlation coefficients for the MOTWSM and the other variables. The correlation of each subscale with the total score of the MOTWSM was in the moderate range: Personal Statement Question One (r = .69, *p* < .01); Personal Statement Question Two (r = .77, *p* < .01); Resume (r = .77, *p* < .01); Letter of Reference One (r = .45, *p* < .01); Letter of Reference Two (r = .45, *p* < .01). Construct validity, the correlation between scores on the MOTWSM and pre-GPA, were not statistically significant (r = 0.23, *p*>.05). The correlation between scores on the MOTWSM and the current method of scoring personal profile packages, was fair (r = .55, *p* < .01).

### Phase 3 reliability and validity

The average time to rate the MOTWSM for a single written submission using the horizontal scoring method was 12 min. The average time was calculated using the data from inter-rater reliability and discriminative validity data. The Cronbach’s alpha coefficient between the items of each subscale ranged from 0.64 to 0.91 and the total MOTWSM had a Cronbach’s alpha coefficient of 0.86.

The results of the two-way random effects ANOVA for the 15 written submissions evaluated to determine inter-rater consistency are presented in Table 3. The intra class correlation coefficients (ICC) for the total subscale scores and total MOTWSM score are above 0.90 (*p*<0.001). ICC for the items on the subscales of the written submissions evaluated for inter-rater consistency ranged from 0.78 to 1.00, with only two items having adequate correlation and the remaining having excellent correlations.

**Table 3.** ICC for inter-rater consistency (IRC) and inter-rater reliability (IRR) for each item, total subscale and total MOTWSM.

	ICC for IRC (n=10)	ICC for IRR (n=75)
PS Q1 Item 1	.854***	.832****
PS Q1 Item 2	.940****	.850****
PS Q1 Item3	.889***	.951****
PS Q1 Item 4	.916****	.844****
PS Q1 Item 5	.867***	.788****
PS Q1 SubscaleTotal	.946****	.908****
PS Q2 Item 1	.905***	.838****
PS Q2 Item 2	.887***	.846****
PS Q2 Item 3	.928****	.931****
PS Q2 Item 4	.887***	.841****
PS Q2 Item 5	.834**	.806****
PS Q2 Subscale Total	1.000	.928****
RESUME Item 1	.941****	.773****
RESUME Item 2	.780*	.850****
RESUME Item 3	1.000	.994****
RESUME Item 4	1.000	1.000
RESUME Item 5	1.000	1.000
RESUME Subscale Total	.971****	.960****
LOR1 Item 1	.968****	.683****
LOR1 Item 2	.919***	.864****
LOR1 Item 3	1.000	.902****
LOR1 Item 4	.957****	.930****
LOR1 Item 5	.984****	1.000
LOR1 Subscale Total	.989****	.940****
LOR2 Item 1	.826***	.889****
LOR2 Item 2	.809***	.918****
LOR2 Item 3	.780*	.934****
LOR2 Item 4	.918***	.932****
LOR2 Item 5	1.000	1.000
LOR2 Subscale Total	.942****	.968****
Total MOTWSM	.984****	.959****

Note: *p< 0.05; **p<0.01; *** *p*<0.005; ****p<0.001; *p*-values are not computed when ICC = 1.000. PS Q1 = Personal Statement Question 1; PS Q2 = Personal Statement Question 2; LOR1 = Letter of Reference 1; LOR2 = Letter of Reference 2.

The two-way random effects ANOVA results for the 75 written submissions evaluated to determine inter-rater reliability are presented in Table 3. The internal consistency across the total subscale scores and the total MOTWSM score for inter-rater reliability are above 0.90 (*p*<0.001). Item correlations ranged from adequate to excellent, with 22 of the 25 items having excellent correlations. The three items with adequate correlations were found in the personal statement question 1 (ICC = 0.79, *p*<0.001), the resume (ICC = 0.77, *p*<0.001), and the letter of reference 1(ICC = 0.68, *p*<0.001).

To test discriminant validity, a one-way ANOVA with planned contrasts was completed (F(2, 192) = 14.43, *p*<0.05, see Table 4). The contrasts identified a significant difference between the applicants that were admitted and waitlisted and those who were admitted and not admitted. However, no significant difference was found in the mean total MOTWSM score of the applicants waitlisted and not admitted.

**Table 4.** Descriptive statistics of discriminative validity data.


Group	n	Minimum	Maximum	Mean	Standard. Deviation
Admitted	65	42	86	65.98^a^	9.239
Waitlisted	65	36	81	58.68^b^	11.495
Not Admitted	65	20	84	55.12^b^	14.032
Total	195	20	86	59.93	12.540

*Note.* F (2, 192) = 14.432, *p*<.001. Non-overlapping superscripts indicate significant group differences.

## Discussion

Research supports the use of reliable and valid admissions measures in admissions processes (Coates, 2008; Dore et al., 2006; Lyons et al., 2006; Salvatori, 2001). Non-cognitive criteria such as written statements, resumes, and letters of reference are commonly used (Bandiera et al., 2015; Thomas et al., 2017) to assess applicants’ potential for future success in health care professions, but psychometrically sound methods of evaluation are limited. With multiple raters often involved in the evaluation of applicants, it is essential that a measure with excellent inter-rater reliability is utilized (Gafni et al., 2003; Salvatori, 2001). The psychometric properties of the MOTWSM support its use in the admission processes to evaluate non-cognitive elements of the submission package.

The findings of this study suggest that the MOTWSM has many good psychometric properties that contribute to the evaluation of non-cognitive criteria of Master of Science in Occupational Therapy admission applications. The measure demonstrated good internal consistency, test-retest reliability, and inter-rater reliability. The 25-item MOTWSM could be completed in an average of 12 min using the horizontal scoring method and was shown to be a concise, easy to use and comprehensive measure.

Findings suggest the measure may work best when considering its total score. When analyzed as a complete measure, the MOTWSM demonstrated strong test-retest reliability (ICC = 0.95), internal consistency (α = 0.76) and inter-rater reliability (α= 0.86). The lower internal consistency coefficients for three subscales, specifically the personal statement question 1, the resume and the letter of reference 1 indicate that raters should complete the entire measure required to obtain a complete representation of an applicant’s written submission. The correlation between scores on the MOTWSM and pre-GPA, were not statistically significant. These results indicate that using pre-GPA alone as an admissions measure may not capture important non-cognitive characteristics of admission candidates. This non-significant correlation indicates that these two constructs are distinct. The inclusion of written statements enhances objectivity when used in the admissions process. Although previous research has shown that pre-course GPA is a strong predictor of academic success, it does not necessarily predict practicum success or those who would become highly effective practitioners. Further research is recommended. Cronbach’s alpha for short scales such as those reported for the MOTWSM subscales are often lower than for longer scales. Further development on the measurement scales could be considered. Additionally, the interpretation of the reported alpha coefficients with these shorter subscales which were in the low range (0.36–0.58) as previously described by Law et al. (2017) need to be interpreted with caution and future research using mean-item correlations could be conducted.

Additional support to illustrate that the MOTWSM is most effective when administered as a complete measure was demonstrated by its excellent inter-rater reliability coefficients (ICC =0.959, *p*<0.001) for total subscale scores and total MOTWSM scores. The high correlations between raters are also good indicators of the measure’s reliability when using scoring guidelines. The scoring guidelines were designed to provide very specific criteria to enable individuals to reliably use the MOTWSM. Therefore, this study provides increased confidence in the ability of a rater to rate the application accurately and reliably using the MOTWSM when applying the scoring guidelines.

The findings of discriminative validity between the non-cognitive elements of the MOTWSM and the current measure demonstrate that the MOTWSM is capturing additional elements, leading to increased objectivity. The one-way ANOVA with planned contrasts identified a significant difference between the applicants that were admitted and waitlisted and those who were admitted and not admitted. However, no significant difference was found in the mean total MOTWSM score between the applicants waitlisted and not admitted. One researcher rated 44 de-identified profiles using the MOTWSM, however, multiple professors and clinicians scored the de-identified profiles using the current measure. Often programs use multiple reviewers to evaluate profile packages and they may be looking for different elements within the written submission measure and this may contribute to increased variability between scores. An objective measure such as the MOTWSM with clear criteria and instructions for scoring non-cognitive criteria could be considered.

The MOTWSM was able to discriminate between the applicants admitted, waitlisted, and not admitted better than the current measure. More specifically, the applicants who were admitted had total MOTWSM scores that were significantly higher than the applicants who were waitlisted or not admitted. Applicants admitted had total MOTWSM scores that were more tightly situated around the group mean, compared to the total MOTWSM scores for the applicants waitlisted and not offered admissions. This indicates that there is less variability in the scores of applicants admitted, and that there is significantly more variability in the scores of the applicants in the other two groups. Therefore, the MOTWSM adds objectivity to the evaluation of the non-cognitive element of the admissions review process. Ultimately, it has the potential to identify the applicants that are most suitable be admitted to the occupational therapy program.

The horizontal scoring method used by each rater when evaluating 75 written submission packages showed some early indications that the halo effect was minimized which is supported by previous research of Dore et al. (2006) & Peeters et al., 2015. This method warrants further investigation for consideration as an effective method of scoring. Furthermore, the mean duration required to complete the evaluation of a written submission utilizing the horizontal scoring method was 12 min and the time to score each profile package decreased as familiarity with the scale increased. These factors illustrate that the MOTWSM is a time efficient measure that is easy to complete and could be considered in the evaluation of applicants to health profession programs.

As previously reported, the evaluation of written statements in admission processes has been unstructured, varies from program to program, and the reliability and validity could be improved by utilizing a standardized Likert scale (Brown et al., 1991). The newly developed MOTWSM with its 25-items and 5-point Likert scale shows promising psychometric properties to enhance the evaluation of the non-cognitive components of admission processes.

Although the findings from the study are specific to an occupational therapy program, many similar qualities and traits of health professionals are evaluated such as communication skills, professionalism, fit, as well as knowledge of the health profession. Health profession programs evaluating written submission packages including statements, resumes and reference letters can consider this measure. Although the study focused on a graduate program, many similar components of the MOTWSM are used as part of the admissions processes in occupational therapy undergraduate programs such as personal statements, resumes and reference letters. Revisions to the MOTWSM’s instructions for evaluation could include relevant high school job experiences, volunteer work and participation in extra-curricular leadership activities. The publications and research presentations section could be revised to include relevant presentations.

In the future, a prospective study could be conducted by further evaluating the MOTWSM with current students in the program. Additionally, other health professional programs that include written submission packages could use and further evaluate the psychometric properties of this measure. Thus, it has the potential for further study across other health professions admission processes. The evaluation of the psychometric properties in diverse populations is important to ensure diversity within the profession. It is crucial that those working in health professions reflect the demographics of the populations that they serve. A longitudinal study would be beneficial to evaluate the effectiveness of the MOTWSM as it relates to program completion, final grade point averages, students’ practicum success, employment status and leadership opportunities throughout their career. Further data analyses are recommended to determine construct validity through factor analysis or Rasch analysis. Further analyses on convergent validity and underlying latent factors are also recommended.

This study has contributed to research evidence on objective admission measures through the development and use of the MOTWSM as a structured admissions measure that can identify the most suitable applicants. The psychometric properties of the MOTWSM support the use of this measure in admission processes.

### Limitations

Limitations of this study include a targeted sample size, the presence of only two raters, items are based on the existing program’s admissions questions, as well as the interpretation of correlations for short scales. The targeted study sample consisted of applicants from one Canadian University occupational therapy program; thus, the results may not be generalizable to all institutions. The two raters within this study do not reflect the entire population of raters that participate in the admissions evaluation process. The use of mean inter-item correlations as an alternative to Cronbach’s alpha was not performed and is considered a limitation of this study. Reported Cronbach’s alphas for short scales in the moderate range need to be interpreted with caution.

## Conclusion

This study demonstrated that the MOTWSM is a reliable and valid measure to evaluate non-cognitive elements of admission applications. The evaluation of non-cognitive criteria is of great value and requires robust and psychometrically sound methods to determine the highest caliber applicants to health care programs. The MOTWSM is a concise, comprehensive measure that has strong psychometric properties. Implementation of the horizontal scoring method may improve the reliability and validity of the MOTWSM and warrants further investigation. Although the evaluation of written submission packages has been questioned for their subjectivity, the MOTWSM, is an objective method that admission committees can confidently utilize in their important decisions surrounding the selection of the most highly qualified applicants to their programs.

## Key findings

• The measure demonstrated good internal consistency, test-retest reliability, and inter-rater reliability.
• The Masters of Science of Occupational Therapy Written Submission Measure is a reliable and valid non-cognitive admissions measure.

## What the study has added

This study provided evidence that a psychometrically sound measure can evaluate the non-cognitive criteria of admissions applications to facilitate selection of the highest caliber of applicants.
